# Rheumatoid factor is correlated with disease activity and inflammatory markers in antineutrophil cytoplasmic antibody-associated vasculitis

**DOI:** 10.1186/s12865-017-0234-8

**Published:** 2017-12-20

**Authors:** Shinji Watanabe, Takahisa Gono, Kumiko Nishina, Naohiro Sugitani, Eri Watanabe, Hiroki Yabe, Chihiro Terai

**Affiliations:** 1 Department of Rheumatology, Saitama Medical Center, Jichi Medical University, 1-847 Amanuma-cho, Omiya-ku, Saitama City, Saitama 330-0834 Japan; 2 Department of Allergy and Rheumatology, Nippon Medical School Graduate School of Medicine, Tokyo, 113-8603 Japan

**Keywords:** Rheumatoid factor, Antineutrophil cytoplasmic antibody, Vasculitis, Disease activity

## Abstract

**Background:**

Some patients with antineutrophil cytoplasmic antibody (ANCA)-associated vasculitis (AAV) also have positivity of rheumatoid factor (RF). However, the clinical significance of this occurrence remains unknown in AAV patients. The aim of this study was to clarify an association between the presence of RF and clinical features in patients with AAV.

**Results:**

Forty-seven patients diagnosed with AAV who were not complicated with RA were enrolled in this study. We compared clinical manifestations of AAV between an RF-positive subset (*n* = 29) and an RF-negative subset (*n* = 18). The Birmingham Vasculitis Activity Score (BVAS) was higher (*P* = 0.026) in the RF-positive subset than in the RF-negative subset. The levels of CRP and ESR were higher in the RF-positive patients (*P* = 0.020 and *P* = 0.007, respectively) compared to the RF-negative subset. IgM-RF titers were significantly correlated with the BVAS (*r* = 0.50, *P* = 0.0004). In addition, the IgM-RF titers had significant correlations with the levels of CRP (*r* = 0.41, *P* = 0.004), ESR (*r* = 0.39, *P* = 0.016), IgM (*r* = 0.36, *P* = 0.016) and IgG (*r* = 0.37, *P* = 0.015). The frequency of commencement of dialysis therapy, usage of mechanical ventilation and mortality were higher in the RF-positive subset than in the RF-negative subset.

**Conclusions:**

In patients with AAV, RF titers were significantly correlated with disease activity and the levels of inflammatory markers. The presence of RF could be a poor prognostic factor in patients with AAV.

## Background

Antineutrophil cytoplasmic antibody (ANCA)-associated vasculitis (AAV) is characterized by necrotizing vasculitis, predominantly affecting small vessels associated with myeloperoxidase (MPO)-ANCA or proteinase 3 (PR3)-ANCA according to the Chapel Hill Consensus Conference (CHCC2012) [1]. AAV includes eosinophilic granulomatosis with polyangiitis (EGPA), granulomatosis with polyangiitis (GPA), microscopic polyangiitis (MPA) and renal-limited vasculitis (RV). Higher doses of corticosteroids with immunosuppressant agents such as cyclophosphamide, methotrexate or mycophenolate mofetil have been administered for remission induction [2–5], and lower doses of corticosteroids with methotrexate or azathioprine have been administered for remission maintenance [5, 6]. Recently, clinical efficacy of rituximab was reported for both remission induction and remission maintenance [5, 7–10].

In a European study, the 5-year survival rate was reported to be 74-91% in patients with GPA, 45-76% in patients with MPA and 45-76% in patients with EGPA. Clinicians need to consider the prognosis when making decisions on the treatment of patients with AAV to control disease activity and promptly prevent irreversible damage. The Five-Factor Score has proven to be useful for evaluating disease prognosis in patients with AAV [11]. Recently, it has been reported that several biomarkers including CRP, C3a, C5a, and IL-18BP in the blood and MCP-1 and C5a in the urine could be useful for the evaluation of disease activity [12].

The rheumatoid factor (RF) is defined as an autoantibody directed against the Fc portion of immunoglobulin G (IgG). RF testing in patients with rheumatoid arthritis (RA) has a sensitivity of 60% to 90% and a specificity of 85% [13]. In infectious diseases and other connective tissue diseases such as Sjögren’s syndrome, systemic lupus erythematosus and polymyositis/dermatomyositis, RF is occasionally detected. In daily practice, up to 37-50% of patients with AAV also have the presence of RF [14]. However, the clinical significance of RF-positivity remains unknown in patients with AAV. Therefore, this study was conducted to clarify an association between the presence of RF and clinical features in patients with AAV.

## Methods

### Patients

This study was retrospectively conducted at Saitama Medical Center, Jichi Medical University. Eighty-one patients were consecutively diagnosed with AAV from January 2006 to December 2015 in our hospital. Among those 81 patients, forty-seven patients with AAV who were not complicated with RA and in whom RF was measured before initiation of treatment for AAV were enrolled in this study. This study was approved by the ethics committee of Saitama Medical Center, Jichi Medical University (approval number 15-62) and was conducted in accordance with the Declaration of Helsinki.

### Diagnosis

Patients with AAV were classified using the European Medicines Agency (EMEA) vasculitis classification algorithm [7]. AAV included EGPA, GPA, MPA and unclassifiable vasculitis (UV). Patients with UV who had the presence of MPO-ANCA or PR3-ANCA were included in this study.

### Data collection

All the data were collected retrospectively from medical records. Information on age, sex, disease duration at diagnosis of AAV, organ involvement, initial dose of prednisolone (PSL), and administration of immunosuppressant agents was retrieved. The disease activity of AAV at diagnosis was evaluated by the Birmingham Vasculitis Activity Score (BVAS) version 3 [15]. Recurrence was defined as an appearance of new or recurrent vasculitis-associated symptoms with active inflammation. Information on commencement of dialysis therapy, usage of mechanical ventilation, mortality and cause of death was also retrieved.

### Laboratory markers

MPO-ANCA and PR3-ANCA were measured using chemiluminescent enzyme immunoassay until November 2012 and enzyme-linked immunosorbent assay starting in December 2012. IgM-RF was measured using a latex agglutination assay. The upper limit of normal was <15 U/mL. Other laboratory markers measured in a usual manner were as follows: hemoglobin, serum albumin, serum creatinine, estimate glomerular filtration rate (eGFR), lactate dehydrogenase, C-reactive protein (CRP), erythrocyte sedimentation rate (ESR), ferritin, IgG, IgM, IgA, complements (C3: [normal range 65-135 mg/dl] and C4: [normal range 13-35 mg/dl]). Anti-cyclic citrullinated peptide (CCP) antibody was also measured using chemiluminescent enzyme immunoassay if sera were available.

### Statistical analyses

Statistical analyses were performed using Fisher’s exact test to compare frequencies and the Wilcoxon rank-sum test to compare median values. Correlation coefficients were established using Spearman’s correlation coefficient. The data were analyzed using JMP software (SAS Institute, NC, USA). A two-tailed *p*-value of <0.05 was considered statistically significant in all analyses.

## Results

### Clinical characteristics of AAV patients at diagnosis

Table 1 shows the clinical characteristics of AAV patients (*n* = 47) at diagnosis. The median (interquartile range [IQR]) age was 67 years (61-76 years). A total of 30 females (64%) participated in the study. The median disease duration at diagnosis was 106 days. EGPA, GPA, MPA, and UV were diagnosed in 10 (21%), 14 (30%), 16 (34%) and 7 (15%) patients, respectively. The positivity of MPO-ANCA and PR3-ANCA was found in 39 (83%) and 3 (6%) patients, respectively. In regard to disease activity associated with AAV, the median (IQR) BVAS was 14 (9-18). The ear/nose/throat, chest, renal and nervous systems were frequently involved in patients with AAV.

**Table 1:** Clinical characteristics of AAV patients at diagnosis

	All of patients (*n* = 47)	RF-positive (*n* = 29)	RF-negative (*n* = 18)	*P* ^*^
Age, years	67 (61-76)	66 (61-73)	67 (58-80)	0.79
Female, no (%)	30 (64)	18 (62)	12 (67)	1
Disease duration at diagnosis, days	106 (42-231)	87 (44-263)	138 (37-232)	0.56
EGPA, no (%)	10 (21)	8 (28)	2 (11)	0.28
GPA, no (%)	14 (30)	7 (24)	7 (39)	0.34
MPA, no (%)	16 (34)	9 (31)	7 (39)	0.75
UV, no (%)	7 (15)	5 (17)	2 (11)	0.69
Positivity of MPO-ANCA, no (%)	39 (83)	22 (76)	17 (94)	0.13
Positivity of PR3-ANCA, no (%)	3 (6)	1 (3)	2 (11)	0.69
BVAS	14 (9-18)	14 (12-22)	12 (6-16)	0.026
Presence of disease activity				
General, no (%)	24 (51)	18 (62)	6 (33)	0.075
Arthralgia/Arthritis, no (%)	7 (15)	4 (14)	3 (17)	1
Cutaneous, no (%)	10 (21)	8 (28)	2 (11)	0.28
Mucous membranes/Eyes, no (%)	2 (4)	2 (7)	0 (0)	0.52
Ear/Nose/Throat, no (%)	19 (40)	11 (38)	8 (44)	0.76
Chest, no (%)	25 (62)	17 (59)	8 (44)	0.38
Pulmonary fibrosis, no (%)	6 (13)	4 (14)	2 (11)	1
Cardiovascular, no (%)	2 (4)	1 (3)	1 (6)	1
Abdominal, no (%)	2 (4)	2 (7)	0 (0)	0.52
Renal, no (%)	17 (36)	9 (31)	8 (44)	0.37
Nervous system, no (%)	23 (49)	20 (69)	3 (17)	0.001
Laboratory data				
Serum albumin, g/dL	3.5 (2.7-3.8)	3.2 (2.6-3.8)	3.8 (3.0-4.2)	0.067
Serum creatinine, mg/dl	0.7 (0.5-1.4)	0.6 (0.5-1.0)	0.7 (0.6-2.2)	0.043
eGFR, ml/min/1.73 mm^3^	71 (33-94)	81 (57-98)	59 (18-81)	0.028
Hemoglobin, g/dl	11.8 (9.7-13.1)	11.8 (10.0-13.1)	12.1 (9.4-13.1)	0.84
C3, mg/dl	106 (86-127)	120 (103-141)	96 (86-125)	0.40
C4, mg/dl	30 (23-38)	32 (21-43)	27 (24-32)	0.36

Values of ages, disease duration, BVAS and laboratory data are expressed as medians (interquartile range). *Statistical analyses were performed using Fisher’s exact test to compare the frequencies and the Wilcoxon rank-sum test for comparisons of median values between the RF-positive subset and the RF-negative subset

*AAV* antineutrophil cytoplasmic antibody (ANCA)-associated vasculitis, *RF* rheumatoid factor, *EGPA* eosinophilic granulomatosis with polyangiitis, *GPA* granulomatosis with polyangiitis, *MPA* microscopic polyangiitis, *UV* unclassifiable vasculitis, *MPO* myeloperoxidase, *PR3* proteinase 3, *BVAS* Birmingham vasculitis activity score

### Comparison of clinical characteristics at diagnosis between RF-positive and RF-negative AAV patients

The positivity of IgM-RF was found in twenty-nine patients with AAV. In these 29 RF-positive patients, the median titer (IQR) of RF was 70 U/ml (27-207 U/ml). We divided the 47 patients into two subsets, RF-positive patients (*n* = 29) and RF-negative patients (*n* = 18). We compared clinical characteristics at diagnosis between the two subsets, as shown in Table 1. There were no significant differences in age, gender, classification of AAV and positivity of ANCA between the two subsets. The double positivity of both MPO-ANCA and PR-3 ANCA was found in 2 RF-negative patients: one with GPA and the other with UV. On the other hand, there were no RF-positive patients with the double positivity of ANCAs. In contrast, the double negativity of both MPO-ANCA and PR-3 ANCA was found in six RF-positive patients and one RF-negative patient. All of these patients with the double negativity of ANCAs were diagnosed with EGPA. Anti-CCP antibody was measured in 14 patients: 10 RF-positive and 4 RF-negative patients, and all of them were negative. There were 2 patients complicated with scleroderma among RF-positive AAV patients: one with anti-centromere antibody and the other with anti-topoisomerase antibody, respectively. On the other hand, there was only one scleroderma patient without any scleroderma-specific autoantibodies among RF-negative AAV patients.

**Fig. 1:**
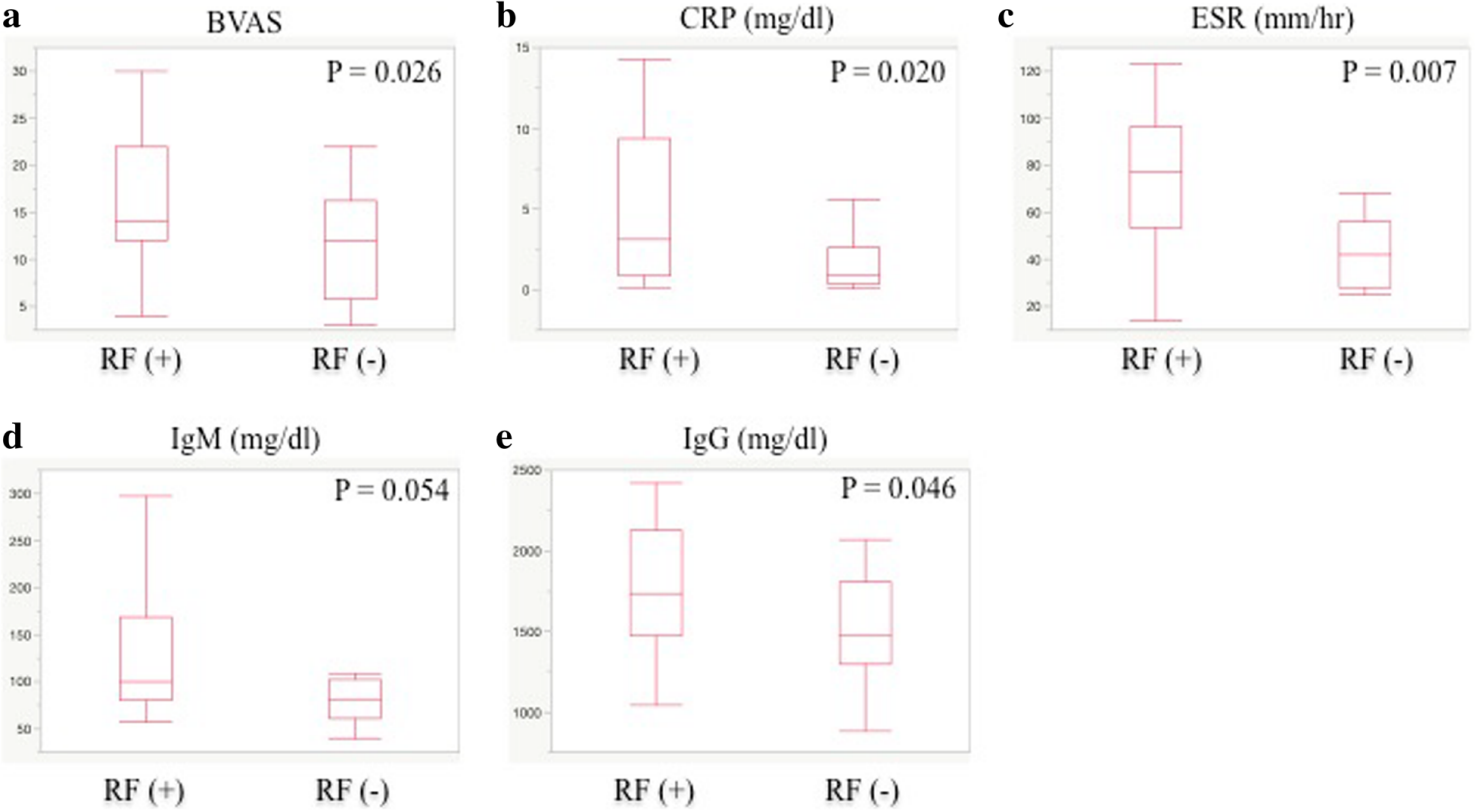
Comparison of disease activity (**a**: BVAS) and laboratory markers (**b**: CRP, **c**: ESR, **d**: IgM and **e**: IgG) between RF-positive AAV patients and RF-negative patients with AAV

BVAS was higher (*P* = 0.026) in the RF-positive patients than in the RF-negative patients (Fig. 1a). Regarding arthralgia/arthritis and lung fibrosis, there were no significant differences between the two subsets. The nervous system was more frequently (*P* = 0.001) involved in the RF-positive patients. According to the levels of serum creatinine and eGFR, renal function was significantly lower in the RF-negative patients than in the RF-positive patients, although there was no significant difference of the frequency of renal involvement between the two subsets.

The levels of serum albumin were lower, and the levels of CRP and ESR were higher (*P* = 0.020 and 0.007, respectively) in the RF-positive patients than in the RF-negative patients (Fig. 1b and c). Additionally, the levels of serum IgM and IgG were higher (*P* = 0.054 and 0.046, respectively) in the RF-positive patients than in the RF-negative patients (Fig. 1d and e), although the statistical significance was weak. Regarding serum levels of C3 and C4, there were no statistical significances between RF-positive and RF-negative AAV patients, although slightly higher in the RF-positive AAV patients (Table1). Serum levels of complements were not lower than the normal ranges in the two subsets.

### Correlations between IgM-RF titer and disease activity in patients with AAV

**Fig. 2:**
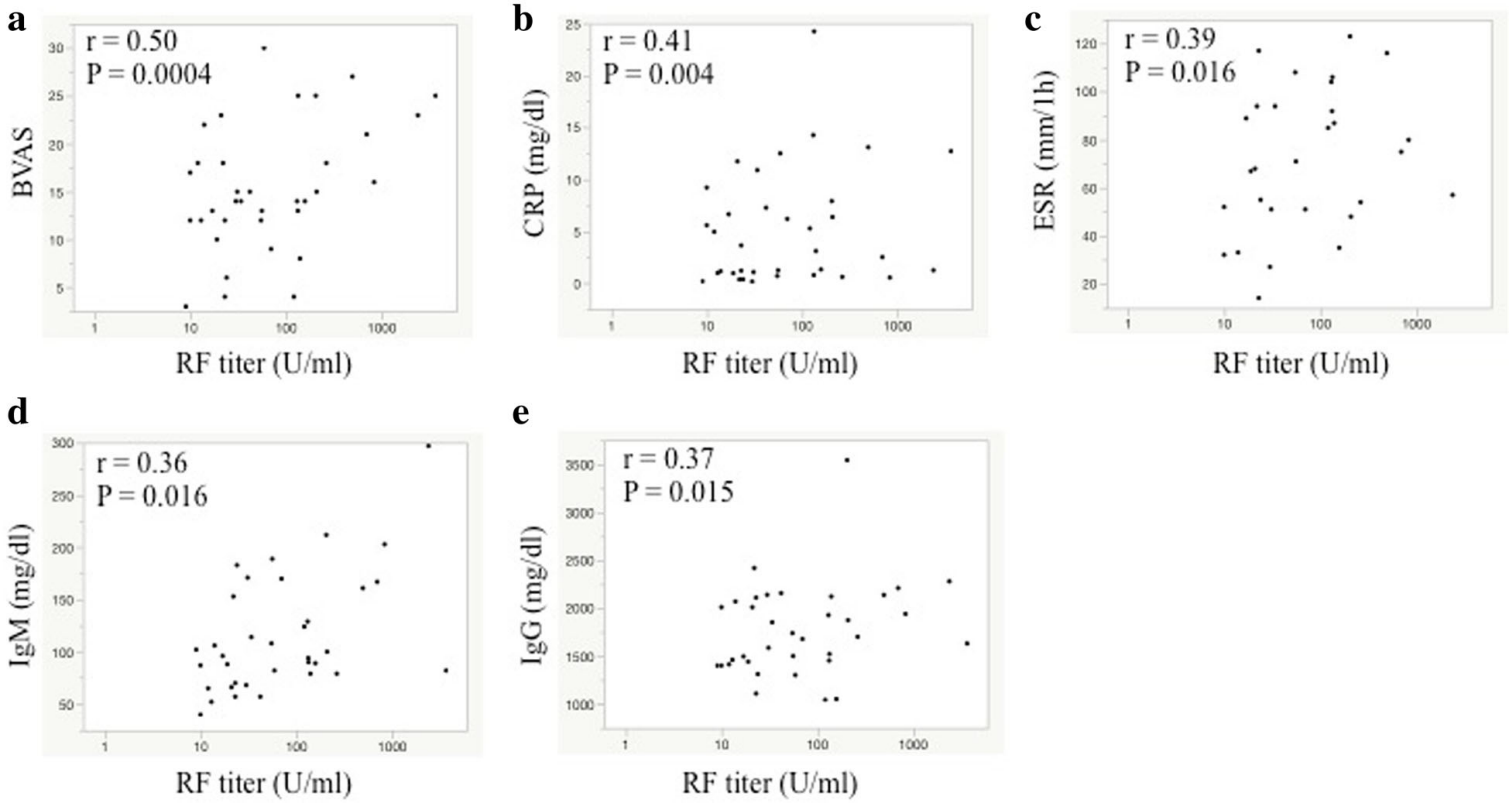
Correlation between RF titers and disease activity (**a**: BVAS) or laboratory markers (**b**: CRP, **c**: ESR, **d**: IgM and **e**: IgG) in patients with AAV

A correlation between the IgM-RF titer and the BVAS is shown in Fig. 2a. IgM-RF titers were significantly correlated with disease activity in all patients with AAV (*r* = 0.50, *P* = 0.0004). In regard to each case of AAV, the IgM-RF titers significantly correlated with disease activity in patients with EGPA (*r* = 0.72, *P* = 0.019), GPA (*r* = 0.64, *P* = 0.014) and MPA (*r* = 0.66, *P* = 0.0059).

### Correlations between IgM-RF titers and laboratory markers in patients with AAV

Serum CRP was the most significant laboratory marker that correlated with the IgM-RF titers (Fig. 2b). In addition, ESR, IgM and IgG had a significant correlation with the IgM-RF titers (Fig. 2c–e). The MPO-ANCA and PR3-ANCA levels did not have a significant positive correlation with RF titers (*r* = 0.18, *P* = 0.24 and *r* = −0.21, *P* = 0.17, respectively).

### Comparison of treatment and prognosis between RF-positive AAV patients and RF-negative AAV patients

The comparison of the treatment content and prognosis is shown in Table 2. For the induction therapy, the initial dose of PSL was higher in the RF-positive subset compared to the RF-negative subset, and the combination therapy of PSL with IVCY was more frequently used in the RF-positive AAV subset than in the RF-negative AAV subset, although there were no statistically significant differences. Immunosuppressive agent as an induction therapy was commenced in ten RF-positive patients and four RF-negative patients. IVCY was administered in five RF-positive AAV patients. On the other hand, the remaining 5 RF-positive patients and the 3 RF-negative patients received CY per orally (POCY). The remaining one RF-negative patient received mizoribine. The difference of the induction treatment, whether IVCY or POCY was administered, could depend on disease activity or serious complication of kidney or lung related to AAV. In contrary, recurrence was more frequent in RF-negative AAV patients than RF-positive patients, although there was no significant difference between the two subsets.

**Table 2:** Comparison of prognosis between RF-positive patients and RF-negative patients with AAV

	RF-positive patients (*n* = 29)	RF-negative patients (*n* = 18)	*P*-value
Initial dose of PSL, mg	40 (30-50)	30 (30-40)	0.07
Immunosuppressive agent usage			
Induction therapy, no (%)	10 (34)	4 (22)	0.52
IVCY, no (%)	5 (17)	0 (0)	0.14
POCY, no (%)	5 (17)	3 (17)	1
Maintenance therapy, no (%)	12 (41)	7 (39)	1
Recurrence, no (%)	3 (10)	4 (22)	0.40
Dialysis, no (%)	4 (14)	1 (6)	0.64
Mechanical ventilation, no (%)	4 (14)	0 (0)	0.28
Dead, no (%)	3 (10)	0 (0)	0.28

Statistical analyses were performed using Fisher’s exact test to compare the frequencies

*RF* rheumatoid factor, *PSL* prednisolone, *IVCY* intermittent pulse intravenous cyclophosphamide therapy, *POCY* oral cyclophosphamide therapy

Initiation of dialysis and usage of mechanical ventilation were more frequently demonstrated in the RF-positive AAV patients than in the RF-negative AAV patients, although no significant differences were observed between the two subsets. Three patients died during the follow-up period; the median (IQR) period was 728 days (346-1057 days). All 3 patients were diagnosed with MPA and were RF-positive. The causes of death were MPA-itself, pulmonary aspergillosis and intra-abdominal hemorrhage, respectively for each of the 3 patients. All of the RF-negative patients remained alive during the follow-up period.

## Discussion

We demonstrated that the titers of RF at diagnosis of AAV had a significant correlation with disease activity in AAV. Additionally, the frequency of initiation of dialysis therapy, usage of the ventilator and mortality were higher in the RF-positive AAV patients than in the RF-negative patients. Therefore, these results suggest that RF could be able to predict prognosis as well as disease activity in AAV. On the other hand, recurrence was more frequent in RF-negative AAV patients than RF-positive patients with no statistical significant difference. This phenomenon might be affected by intensity of treatment. IVCY as an induction therapy was more often administered in RF-positive AAV patients than in RF-negative patients. The difference of treatment content might depend on relapsing rate between the two subsets.

Until now, there have been several studies investigating the relationship between AAV and RA. One previous study reported that RF can be detected in 37-50% in patients with AAV [14]. However, the positivity of anti-CCP antibody is rare. Tervaert et al. reported that among 133 patients with AAV, RF can be detected in thirty-four (26%) patients with AAV, and twenty-eight (82%) RF-positive patients had migratory arthralgia and/or arthritis [16]. However, anti-CCP antibodies were detected in only three patients. None of three patients developed erosive disease during long-tern follow-up [16]. According to another previous report by Pagnoux et al., five P-ANCA-positive AAV patients with anti-CCP suffered from polysynovitis [14]. Articular symptoms preceded AAV diagnoses by a median of five months. Four of five patients fulfilled the ARA/ACR 1987 criteria for RA, although none had radiologically detected destructive arthritis. These results suggest that RA and AAV could be difficult to distinguish in the early phase of disease in AAV patients with RF/anti-CCP-positive [16]. Draibe et al. have reported that there was an overlap with RA in AAV patients, although AAV rarely occurred in RA patients [17]. In our study, anti-CCP antibody was detected in none of AAV patients, although measured in only fourteen patients. Seven (15%) patients including four RF-positive and three RF-negative, had arthralgia/arthritis at diagnosis of AAV. There might be some of AAV patients overlapping RA in our study.

The RF is an autoantibody directed against the Fc portion of immunoglobulin G (IgG). IgM-RF is the predominant subtype in RF and is commonly measured in clinical practice for the diagnosis of RA. The RF can be detected in non-RA diseases such as infectious diseases and connective tissue diseases [13]. The RF revealed during infections is usually considered transient and not pathogenic for arthritis [13, 18]. This type of RF is different from the RF found in RA [18, 19]. The RFs found in infections have a low affinity and are multi-reactive for microorganisms and are not strongly involved in the development of RA. In contrast, the RF found in RA has a high affinity. The low-affinity RF could be involved in a clearance of immune complexes (ICs) composed of microorganisms and IgG as a host defense to infections [13, 20]. Moreover, RF is more frequently revealed in elderly people without RA, ranging from 1.3% to 4% in the elderly population [18, 21, 22]. This phenomenon might be attributed to age-related immune deregulations. In our study, IgM-RF was measured repeatedly in 7 RF-positive AAV patients. IgM-RF titers got decreased after induction therapy in 6 patients, and reached the normal range in 4 patients. In only one patient, the high titers sustained. Therefore, RF positivity could be transient in AAV, although immunosuppressive treatment could affect the titers. Therefore, the RF found in AAV could be the low-affinity RF.

The low-affinity RF is produced by CD5-expressing B cells, called B1a-B cells [23], in healthy subjects or patients with infection. The B1a-B cells produce the low-affinity IgM RF as natural IgM antibodies that have an ability to bind multiple antigens such as bacterial lipopolysaccharides and the Epstein-Barr virus including IgG Fc fragments [13, 19, 23]. This IgM production is independent of T cells as innate immunity. In contrast, the high-affinity RFs found in RA are produced by B2-B cells with the necessity of co-stimulation of the antigen-presenting cells and T cells. Although it has remained unknown how the positivity of RF is associated with the pathophysiology of AAV, B1a-B cells might be activated by infections or some factors associated with AAV such as ANCA. Our study demonstrated that the titers of IgM-RF were significantly correlated with disease activity in AAV. The levels of IgM-RF might reflect the intensity of activated B1a-B cells involved in the disease activity of AAV.

The activation of monocytes as well as neutrophils are involved in the pathophysiology of AAV [24]. Engagement of Fcγ receptors by ICs including ANCA and antigens such as MPO and PR3 on neutrophils or monocytes plays a role in the development of inflammation [24, 25]. A previous study demonstrated that monocyte activation correlated with disease activity in patients with GPA [17]. Proinflammatory cytokines such as interleukin-6 (IL-6) produced by activated monocytes were increased in patients with GPA. Moreover, the levels of IL-6 had a significant correlation with the BVAS in GPA. Our study showed that IgM-RF titers were significantly correlated with CRP and the BVAS. CD14-positive monocyte lineage cells induced by granulocyte-macrophage colony-stimulating factor from bone marrow stimulate the proportion of IgM-RF produced by B cells [18]. Taken together, a cross-link between IgM-RFs and ANCA-including ICs might stimulate the production of inflammatory cytokines/proteins such as IL-6 and CRP by monocyte/macrophage-lineage cells and intense disease activity. In this study, IgM-RF was significantly correlated with BVAS and inflammation markers such as CRP and ESR. More severe complications such as end stage renal disease and pulmonary dysfunction happened in RF-positive AAV patients than in RF-negative AAV patients. These results suggested that IgM-RF is a potent factor associated with activation of inflammation and disease severity.

The main limitations of this study were the small size and the retrospective design of the study. RF was checked in part of patients with AAV. In addition, among 47 patients enrolled at this study, the number of patients with EGPA was 10. The prevalence of EGPA was higher than that usually reported. The percentage of ANCA-positive patients is higher in the RF-negative AAV patients than RF-positive those, although there is not statistically significance. Double negativity of both MPO-ANCA and PR-3 ANCA was found in patients with EGPA. EGPA might be associated with the presence of RF, but less with the presence of ANCAs. These findings could affect selection bias and results in this study. In Japan, the prevalence of MPA is more frequent than that of GPA [19]. There is a little difference in the prevalence between MPA and GPA in this study compared to Western countries.

## Conclusions

RF titers are significantly correlated with disease activity and inflammatory markers in AAV. The presence of RF could be a poor prognostic factor in patients with AAV.

## Abbreviations

AAV: ANCA-associated vasculitis
ANCA: Antineutrophil cytoplasmic antibody
BVAS: Birmingham Vasculitis Activity Score
CCP: Cyclic citrullinated peptide
CRP: C-reactive protein
eGFR: Estimate glomerular filtration rate
EGPA: Eosinophilic granulomatosis with polyangiitis
EMEA: European Medicines Agency
ESR: Erythrocyte sedimentation rate
GPA: Granulomatosis with polyangiitis
ICs: Immune complexes
IgG: Immunoglobulin G
IQR: Interquartile range
MPA: Microscopic with polyangiitis
MPO: Myeloperoxidase
PR3: Proteinase 3
PSL: Prednisolone
RA: Rheumatoid arthritis
RF: Rheumatoid factor
RV: Renal-limited vasculitis
UV: Unclassifiable vasculitis
